# NICU discharge preparation and transition planning: introduction

**DOI:** 10.1038/s41372-022-01312-w

**Published:** 2022-02-14

**Authors:** Vincent C. Smith

**Affiliations:** Boston Medical Center, Boston, MA USA

## Abstract

The National Perinatal Association (NPA) coordinated a multidisciplinary work group to develop guidelines and recommendations for Neonatal Intensive Care Unit (NICU) discharge preparation and thus the transition from NICU to home for infants admitted to the NICU and their families. In this section, we explore the concepts of NICU discharge readiness as well as transition planning and preparation. We describe the process that was used to develop the guidelines and recommendations as well as the timeline for the work. NPA hopes that the readers will find the Interdisciplinary Guidelines and Recommendations for NICU Discharge Preparation and Transition Planning to be beneficial, useful, and pertinent.

## National perinatal association

The National Perinatal Association (NPA), an interdisciplinary organization of professionals, parents, and advocates, is a leading voice in perinatal care in the United States. Our mission is to advocate for pregnant people, infants, families, and the professionals who care for them. NPA works to create positive change in perinatal care by partnering with healthcare, governmental, and nonprofit organizations. NPA is families and professionals working together to inform changes in policy and practice that are based in the evidence and informed by experience. As such, NPA brings all stakeholders to the table with a commitment that all voices are heard equally and respectfully.

NPA coordinated a multidisciplinary work group to develop guidelines and recommendations for Neonatal Intensive Care Unit (NICU) discharge preparation and thus the transition from NICU to home for infants admitted to the NICU and their families. The overarching goal is to develop recommendations for transition from the NICU to home that can be implemented broadly.

## NICU discharge readiness

In 2010, an estimated 11.1% of all live births worldwide (~14.9 million babies) were born preterm (<37—weeks gestation) [1]. The United States is one of the ten countries with the highest numbers of preterm births [1]. Most preterm babies will require some care in a NICU. Other babies will need the specialized care that NICUs offer. Infants who need NICU level of care, preterm and otherwise, will eventually be discharged and their families need preparation and transition planning.

The recommendation from the American Academy of Pediatrics is that the time to transition a family from the NICU to home is after an infant has achieved physiologic stability and the primary caregivers (e.g., in most instances the parents) have participated in an active program of preparation for care of the infant at home [2]; generally in practice, this has translated into the timing of NICU discharge being driven primarily by the physiological maturity of the infant. Another component of discharge planning is to make sure that arrangements for follow-up outpatient care have been completed and that the family has obtained the necessarily skills and education required to care for their baby in their home environment. Without adequate parent education there is an increased risk of readmission because parents may not seek medical attention appropriately, administer medications and other therapies correctly, or have confidence in their home management of non-acute medical problems [2]. Still, there has only been limited guidance offered on what the content of a comprehensive discharge planning program for the family should be [3, 4].

NICU discharge readiness has been previously defined as “the attainment of technical skills and knowledge, emotional comfort, and confidence with infant care by the infant’s primary caregivers at the time of discharge” [4]. Similarly, NICU discharge preparation has been previously defined as “the process of facilitating discharge readiness to successfully make the transition from the NICU to home” [4]. Therefore, the desired outcome is discharge readiness, but the process to get to the desired outcome is discharge preparation [4]. For a NICU discharge preparation program to be comprehensive, it would need to address all of the following:

1. An approach to discharge education.
2. A thorough structured education program.
3. A clearly defined curriculum.
4. An assessment of the discharge readiness of the family.
5. A way to consistently transfer ongoing care to a medical home.

Because there were no national guidelines for this process, the NPA decided to help fill the gap.

## Development of the “Interdisciplinary Guidelines for NICU Discharge Preparation and Transition Planning”

In 2017, NPA convened a work group to develop guidelines for NICU discharge preparation and transition planning. The work group consisted of the chair, the executive team (i.e., NPA’s Executive Director, Director of Communications, and Director of Development), and interdisciplinary members. The NICU discharge preparation and transition planning process began with an environmental scan that reviewed existing standards for NICU discharge preparation. An environmental scan is a process that systematically surveys and interprets relevant data to identify external opportunities and threats that could influence future decisions. The work group collected and collated existing standards for NICU discharge preparation. The work group conducted a literature review of published medical and nursing literature related to the topic. The environmental scan became the basis to draft evidence-based, multidisciplinary, and consensus guidelines on NICU discharge preparation and transition planning over a year-long process. The work group began monthly conference calls where they would work on the guidelines in small sections. During these calls, the work group determined where consensus exists, recognized differences in practice, identified gaps where no guidelines were available, and created a course of action to address these gaps. The environmental scan was periodically updated. By early 2019, the work group drafted, edited, revised, and approved the set of guidelines.

In June 2019, the NPA hosted a national summit with content experts to review the draft guidelines created by the work group. Prior to the conference, the content experts were divided into four topic-based groups (i.e., family and home assessments, special circumstances, support systems, and transfer and/or coordination of care). Each group of content experts were given the following questions to consider as they reviewed the draft guidelines:

1. Is the content appropriate?
2. Is the content complete (i.e., Is there content missing)?
3. Are the recommendations appropriate, practical, and actionable?
4. Are the recommendations clear and concise?
5. Are the recommendations complete?

The summit consisted of sixteen multidisciplinary experts representing fourteen different organizations. During the summit, the content experts reviewed their assigned sections of the guidelines. At the end of the summit, each group of content experts produced a report and devised next steps for the draft guidelines.

Five members of the work group affectionately referred to as the “small group” verified each reference associated with the guidelines from the original article or guidance and assessed the level of evidence for each item. Members of the small group worked in parallel so that each item was reviewed by at least two work group members. When there was not consensus between the two work group members, the chair reviewed the item and made the deciding vote. The small group review was completed by December 2020.

In January 2021, the NPA hosted a second national summit with content experts to review the revised guidelines. The summit consisted of twenty-two multidisciplinary experts representing nineteen different organizations. Similar to the first summit, the content experts were assigned to one of five topic-based groups. During the summit, the content experts reviewed their assigned sections of the guidelines with the same questions for discussion as the first summit. At the end of the summit, each group of content experts produced a report and devised next steps for the draft guidelines. These reports were used to create the final draft of the guidelines.

Members of the “small group” performed a final editorial review of the draft guidelines. The work group’s chair and executive team formatted the guidelines for publication and prepared them for submission to the *Journal of Perinatology*.

NPA hopes that the readers will find the Interdisciplinary Guidelines and Recommendations for NICU Discharge Preparation and Transition Planning to be beneficial, useful, and pertinent. We want these guidelines to help facilitate consistency and efficiency in comprehensive discharge preparation and transition planning for NICU infants and their families. Ideally, these guidelines will assist staff in providing clear and consistent messages of both action and guidance for parents and families, as well as provide a systematic approach to required tasks and advanced planning of discharge teaching prior to anticipated discharge. Our hope is that these guidelines will provide more uniformity in discharge preparation, and consequently, reduce uncertainty and stress with the discharge preparation and transition planning process. Going home from the NICU should be a time of joyous celebration. Hopefully these guidelines will ensure that discharge planning and teaching occur in a timely, organized, and consistent manner, thereby improving family and staff satisfaction as well as patient care.
